# Knee Arthrodesis with a Modular Silver-Coated Endoprosthesis for Infected Total Knee Arthroplasty with Extensive Bone Loss: A Retrospective Case-Series Study

**DOI:** 10.3390/jcm12103600

**Published:** 2023-05-22

**Authors:** Olga D. Savvidou, Angelos Kaspiris, Stavros Goumenos, Ioannis Trikoupis, Dimitra Melissaridou, Athanasios Kalogeropoulos, Dimitris Serenidis, Jim-Dimitris Georgoulis, Ioanna Lianou, Panagiotis Koulouvaris, Panayiotis J. Papagelopoulos

**Affiliations:** 1 Medical School, First Department of Orthopedics, Attikon University Hospital, National and Kapodistrian University of Athens, 12462 Athens, Greece; olgasavvidou@gmail.com (O.D.S.); stgoumenos@gmail.com (S.G.); gtrikoupis@hotmail.com (I.T.); dimitramelissaridi@gmail.com (D.M.); dserenidis21@gmail.com (D.S.); jim.georgoulis@gmail.com (J.-D.G.); info@drkoulouvaris.gr (P.K.); 2 Laboratory of Molecular Pharmacology, School of Health Sciences, University of Patras, 26504 Patras, Greece; angkaspiris@hotmail.com; 3 Sonnenhospital, 3006 Bern, Switzerland; athanasios.kalogeropoulos@gmail.com; 4 Department of Orthopaedics, Rion University Hospital University of Patras, 26504 Patras, Greece; jolianou@hotmail.com

**Keywords:** periprosthetic joint infection, knee arthrodesis, silver- coated endoprosthesis

## Abstract

Introduction: Knee arthrodesis is a limb salvage intervention for persistent periprosthetic joint infection (PJI) when revision total knee arthroplasty fails. Conventional arthrodesis techniques are associated with the increased rate of complications, especially in patients with extensive bone loss and extensor tendon deficiency. Methods: Eight patients with a modular silver-coated arthrodesis implant after failed exchange arthroplasty for infection, were retrospectively reviewed. All patients had significant bone loss, while 5 displayed extensor tendon deficiency. Survivorship, complications, leg length discrepancy, median Visual Analogue Scale (VAS) and Oxford Knee score (OKS) were evaluated. Results: The median follow up was 32 months (range 24–59 months). The survivorship rate of the prosthesis was 86% during the minimum time of follow up of 24 months. In one patient recurrence of the infection was observed and above-knee amputation was performed. The median postoperative leg length discrepancy was 2.07 ± 0.67 cm. Patients were able to ambulate with mild or no pain. The median VAS and OKS was 2.14 ± 0.9 and 34.7 ± 9.3, respectively. Conclusions: The results of our study demonstrated that knee arthrodesis with a silver coated arthrodesis implant, performed for persistent PJI in patients with significant bone loss and extensor tendon deficit, provided a stable construct, allowed eradication of infection and was associated with good functional outcome.

## 1. Introduction

Periprosthetic joint infection (PJI) after Total Knee Arthroplasty (TKA) demonstrates prevalence that ranges from 1% to 2% and is associated with implant failure [1]. In 9% to 12% of cases, the infection may persist despite the removal of the implant and the application of antibiotic spacers [2,3]. Moreover, the incidence of infection after revision arthroplasty for infected TKA is higher, reaching 26% [4]. The eradication of PJI is multifactorial and depends on the type of isolated organism, the chronicity of the infection and the immunologic status of the host. 

Patients with persistent PJI usually have large bone defects, extensor mechanism incompetence and severe soft tissue deficiency, due to multiple implant extractions of failed TKAs and extensive debridement [5,6]. Resection arthroplasty and above knee amputation (AKA) should be considered as end-staged therapeutic options [7]. For patients with multiple comorbidities, a long-term antibiotic suppression regime and a static or articulating antibiotic-loaded cement spacer can offer a painless, semi functional limb [2,8]. The decision to undertake a limb salvage procedure, with no knee motion, instead of a two-stage revision TKA is challenging. 

Different techniques for knee arthrodesis have been reported including intramedullary nails, external fixators and compression plates [9,10,11,12]. Intramedullary nails of different length and modularity are the most used method for knee arthrodesis, which is mainly correlated with good results [13]. However, the reported complication rates were reported to reach 55% [14]. External fixation, which is another method with comparable long term results regarding bone fusion [15,16], is usually complicated with pin track complications in 80% of patients [17]. Although dual plating techniques with locking compression plates (LCP) were associated with increased stability [18], sufficient bone stock and delayed weight-bearing until fusion was required [12]. The most common complications of the above methods were implanting failure and non-union [19,20], while the outcomes of knee arthrodesis following persistent PJI after failed TKA were not as successful as in aseptic TKA and delayed or non-unions were frequently presented [6,10]. Conventional knee arthrodesis methods cannot achieve an adequate apposition of bone and successful fusion was accompanied by significant limb shortening. Autologous cancellous bone grafting, vascularized fibular grafts, allografts and bone transport using circular frames have been used and were linked to good functional outcomes. In addition to advanced demanding surgical skills of these techniques, they are also correlated with high rates of complications, repeated surgical procedures and delayed weight-bearing [21].

Modular knee arthrodesis implants were developed to overcome the disadvantages of the conventional methods. They offer modularity allowing for segmental deficit reconstruction, immediate postoperative stabilization and early weight bearing [21,22]. Silver is used as an antimicrobial element in several implant systems with promising results [23]. According to the results from various in vitro studies, the presence of silver coating can effectively inhibit, or even prevent the formation of biofilms of various bacteria on metal surfaces [23,24,25]. The efficacy of these implants in preventing infections of endoprostheses has been confirmed by clinical studies, too [26,27]. However, literature regarding the outcomes of silver-coated modular knee arthrodesis implants is limited. 

The aim of the present study was to assess the survivorship, complications, limb length discrepancy and patient functional outcomes after knee arthrodesis with a modular silver-coated prosthesis in patients with persistent PJI after TKA and a failure of two-stage revision intervention. 

## 2. Materials and Methods

**Figure 1:**
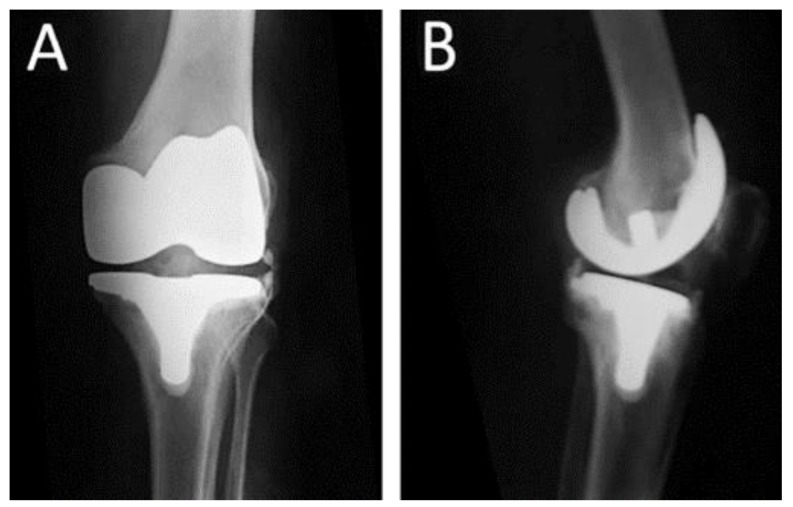
Postoperative radiographs of a 67-year-old female who underwent total knee replacement due to osteoarthritis of the left knee (**A**) Anteroposterior and (**B**). Lateral radiograph of the knee.

**Figure 2:**
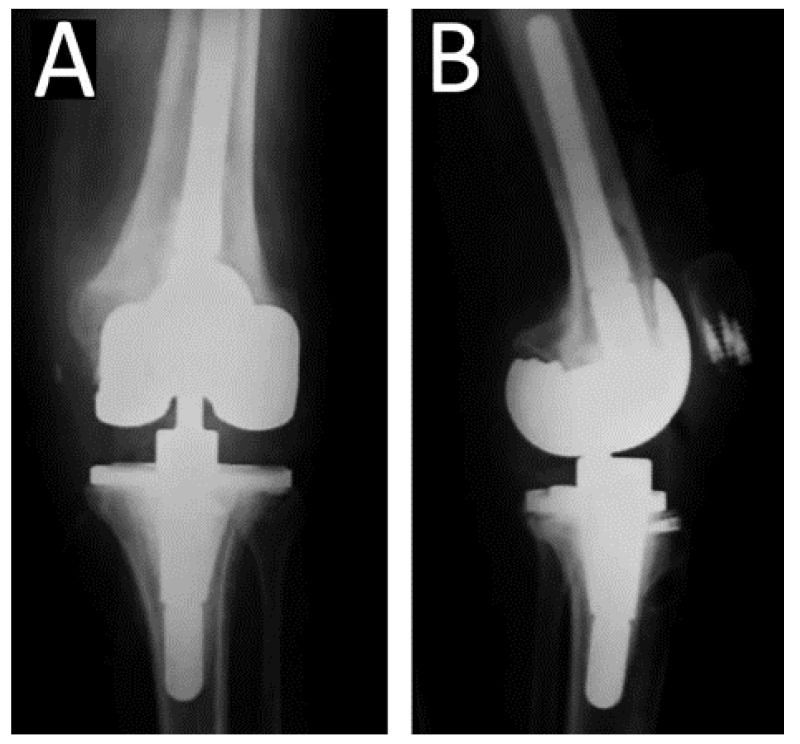
Due to persistent pain and stiffness the patient underwent revision total knee Arthroplasty (CCK) 6 years postoperatively. Due to intraoperative rupture of the patellar tendon, the patellar tendon was augmented with a semitendonous autograft, combined with a posterior tibialis tendon allograft and a synthetic graft jacket. The patellar tendon was sutured with bone anchors at the distal part of the patella. (**A**) Anteroposterior and (**B**) Lateral radiograph of the knee.

The clinical records and imaging studies of 8 consecutive patients with persistent PJI after two-stage revision for infected TKA that underwent knee arthrodesis with a silver-coated modular knee prosthesis (MUTARS^®®^- Munster Arthrodesis Implant cast GmbH, Buxtehude, Germany) between 2016 and 2022 in our department were retrospectively reviewed. Primary osteoarthritis was the indication for primary TKA in all patients (Figure 1 and Figure 2).

**Figure 3:**
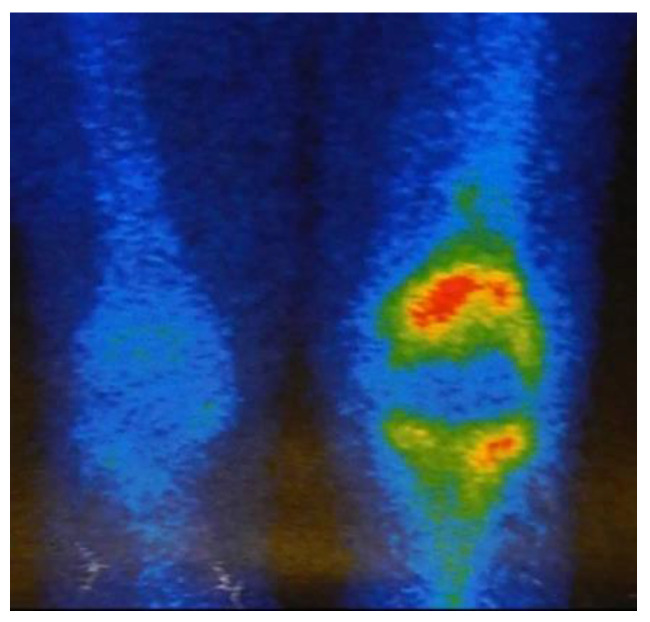
Bone scintigraphy showed an increase in Tc-99 uptake in the left knee joint.

The microorganisms responsible for the knee infections (Figure 3) varied widely and included 4 cases of methicillin resistant Staphylococcus aureus (MRSA), 2 cases of vancomycin-resistant Enterococcus, and 2 cases of multi-organism infections. 

**Figure 4:**
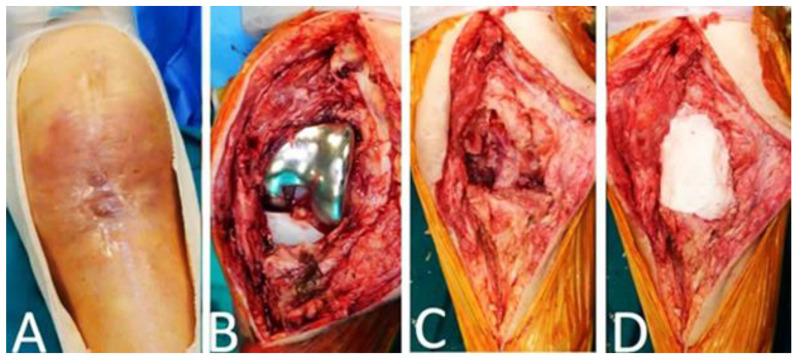
(**A**) Sixteen months after the index operation patient had clinical signs of persistent periprosthetic knee infection. (**B**,**C**) Meticulous debridement, removal of the implants and (**D**) Polymethyl methacrylate (PMMA) coated with gentamicin and vancomycin was applied.

All total knee arthroplasties were removed, and meticulous debridement was performed, followed by insertion of antibiotic-impregnated cement spacers mixed with gentamicin and vancomycin (Figure 4). 

In all patients, intravenous antibiotics were administered for 2-weeks followed by a 6–10 weeks per-os antibiotic regimen based on bacterial cultural results, microbial sensitivity tests and serial evaluation of inflammatory markers. If clinical examination and laboratory tests suggested persistence of infection, further debridement, and the exchange of the antibiotic-loaded polymethyl methacrylate (PMMA) spacer were performed. Before, knee arthrodesis patients had an average of 2.1 procedures for spacer placement and 0.9 irrigation and debridement procedures. Knee arthrodesis was performed only when clinical examination, laboratory tests and knee aspiration were indicative of infection clearance.

The study was compiled with the 1975 Declaration of Helsinki, and it has been approved by the Institution’s Ethical Committee of our Hospital (Registration No: ΕBD 231/19-4-2021).

### 2.1. Technique

**Figure 5:**
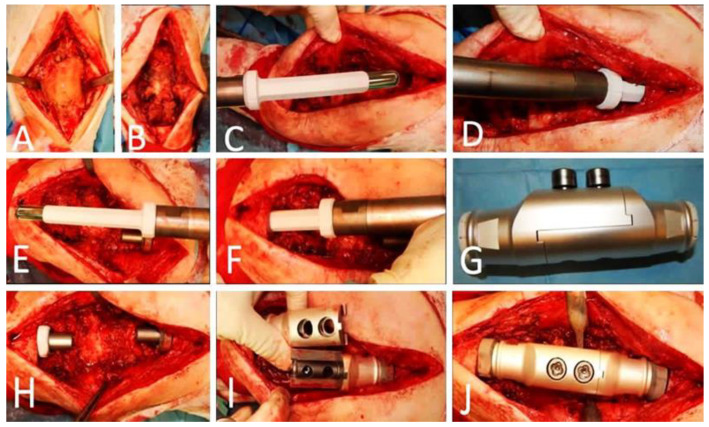
Intraoperative images showing (**A**) The antibiotic-loaded polymethyl meth acrylate (PMMA) spacer with gentamicin and vancomycin. (**B**) Extensive bone loss after extensive debridement and curettage of the canals. (**C**) insertion of the femoral cementless stem. (**D**) impaction of the stem to the femoral canal. (**E**) Insertion of the cementless tibial stem. (**F**) impaction of the stem to the tibia canal. (**G**) the body of the silver coated arthrodesis implant. (**H**) after femoral and tibial stems insertion and extensive bone loss noted, the arthrodesis implant was ready to be inserted. (**I**) insertion of the distal part of the arthrodesis implant to the tibial stem and insertion of the proximal part of the arthrodesis implant to the femoral stem. The proximal and the distal part of the arthrodesis implant were combined. (**J**) the parts of the arthrodesis implant were connected with two locking screws.

All surfaces were meticulously debrided of necrotic tissues with curettage and the intramedullary canals were reamed to remove all infected tissues. Multiple, at least five, intraoperative tissue samples were taken for cultures. After radical debridement, a modular silver-coated knee arthrodesis prosthesis (MUTARS-Munster Arthrodesis implant cast, Buxtehude, Germany) was used for reconstruction. The minimum resection length of this implant system is 145 mm (145–265 mm). The system consists of a femoral stem cemented (implavit^®®^ implant cast, Buxtehude, Germany; CoCrMo casting alloy), or cementless (implatan^®®^ implant cast, Buxtehude, Germany; TiAl6V4 with HA coating), an arthrodesis implant (implatan^®®^ implant cast, Buxtehude, Germany; TiAl6V4) with silver coating, a tibial plate (implavit^®®^ implant cast, Buxtehude, Germany; CoCrMo-casting alloy) with silver coating, an extension piece (implatan^®®^ implant cast, Buxtehude, Germany; TiAl6V4) with silver coating and a tibial stem cemented (implavit^®®^ implant cast, Buxtehude, Germany; CoCrMo casting alloy) or cementless (implatan^®®^ implant cast, Buxtehude, Germany; TiAl6V4 with HA-Coating) (Figure 5).

In all eight patients cementless femoral and tibia stems were used. In two out of eight patients (28.6%), due to severely compromised soft-tissue coverage, a medial gastrocnemius flap was used for the coverage of the prosthesis. All surgeries were performed by the same surgeon (senior author P.J.P.).

The diagnosis of PJI was based on the criteria of the International Consensus Group for Periprosthetic Infections. Specifically, two major (two positive periprosthetic cultures of the same microbe or sinus-tract communication with the joint) and five minor criteria (increased serum C-reactive protein, CPR, and erythrocyte sedimentation rate, ECR; increased white blood cells or polymorphonuclear neutrophils or positive leukocyte esterase (LE) strip test for synovial fluid and a positive histological test from periprosthetic tissue sample) should be met. The diagnosis of PJI was set when at least one major and three minor criteria were fulfilled [28]. 

**Figure 6:**
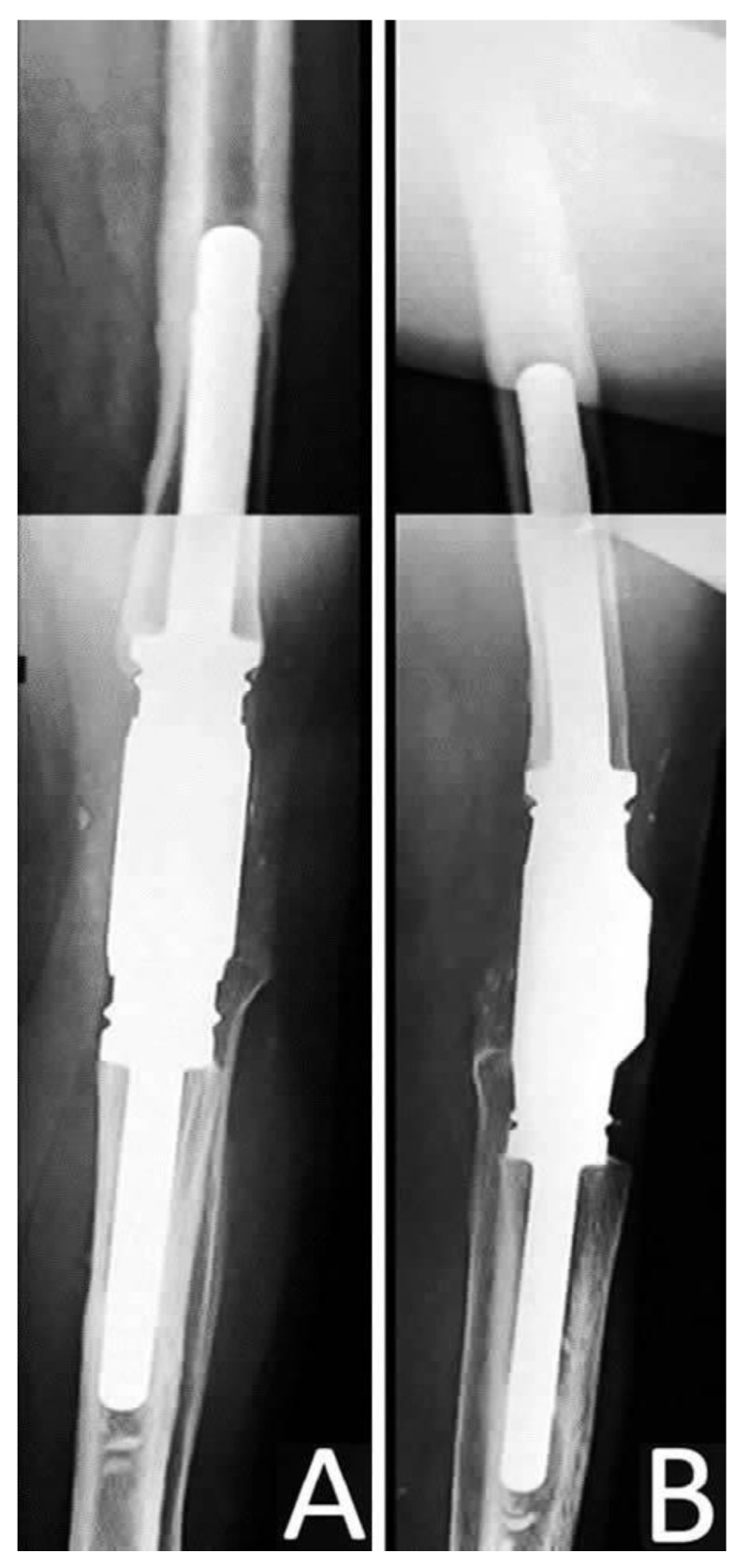
(**A**) Anteroposterior and (**B**) Lateral post-operative radiograph of the knee after the application of the silver-coated endoprosthesis.

The primary endpoint was the outcome (success vs. failure) of the surgical treatment according to prosthesis survivorship during the postoperative period (Figure 6). The secondary endpoint was the assessment of the functional clinical results of the patients at their final follow up examination. The outcome was considered successful if no clinical or microbiological signs of infection were documented following surgical and antibiotic regime within the 24 months postoperative and patients achieved a painless, stable joint. Any other condition was considered as treatment failure [29]. The functional outcome was evaluated using the Visual Analogue Scale (VAS) for postoperative pain assessment and the Oxford Knee Score (OKS) [30,31]. All patients were ambulatory immediately at the first postoperative day with partial weight bearing, while full weight bearing began after 6 weeks.

### 2.2. Statistical Analysis

Categorical data are presented as numbers (percentages), and continuous data are presented as medians, in the text and figures.

## 3. Results

### 3.1. Baseline Characteristics

The baseline characteristics of the patients are shown in Table 1. There were eight patients who were diagnosed with periprosthetic infection after revision of the knee arthroplasty and underwent surgical intervention with modular silver-coated endoprosthesis. There were three males and five females, while the median age and Body Mass Index (BMI) were 67 years (min: 57, max: 75, SD: 6.50) and 27.14 kg/m^2^ (ranging from 22 to 31, SD: 3.24), respectively. All 8 patients had segmental bone loss, while five patients had an extensor mechanism deficiency (Table 1). The median length of bone loss was 172 mm (range, 145–205 mm). Moreover, clinical information recorded at the final follow-up included the absence for evidence of immunosuppression, previous radiotherapy, oncological diseases, lymphoedema, vascular and pulmonary diseases, as well as previous multiple surgical interventions. 

### 3.2. Clinical and Surgical Outcomes

The median follow-up period was 32 months (ranging from 24 to 59 months, SD: 11.88). The survivorship rate of the knee arthrodesis modular silver-coated prosthesis (primary endpoint) was 86% (six out of eight patients) during the minimum time of follow up of 24 months. One patient (14%) developed a periprosthetic infection recurrence and wound dehiscence. This patient underwent an above knee amputation and has remained free of complications for the last 30 months. In two of the patients (28%) one out of eight positive intraoperative cultures (*Staphylococci* sp.) were detected at the final implantation surgery, and intravenous antibiotic therapy was administered for two weeks. During the last follow up these patients did not show any clinical or serological symptoms of infection or implant-associated complications based on Henderson classification. The remaining five patients (71.4%) did not develop infection relapse and implant-associated complications during the follow up period. Postoperative calculation of leg length discrepancy showed that the affected leg was 2.07 ± 0.68 cm shorter. Regarding the secondary endpoints, none of the patients complained about severe pain at their last follow up (median VAS value = 2.4 ± 0.9) and their functional status was very satisfactory (median OKS value = 34.7 ± 9.3). All patients were able to perform everyday activities with occasional use of a cane (Table 1). Finally, clinical signs of argyria, peripheral neuropathy or other silver associated side effects were not observed in any individual. 

## 4. Discussion

The treatment of persistent PJI after TKA is challenging and is associated with increased morbidity and financial burden [32]. Even though two-stage revision TKA is correlated with elevated risk of re-infection, morbidity and mortality, it remains the gold standard for the treatment of chronic PJI [33]. However, when revision or re-revisions in an infected TKA is not a viable option, knee arthrodesis may be considered as a limb salvage procedure [34,35]. Our study examined the functional and clinical outcomes and investigated the restoration of limb length and alignment after the application of modular silver-coated endoprosthesis for the infected knee arthroplasty with extensive bone loss and extensor mechanism deficiency. An additional objective of this study was to evaluate the rate of persistent or recurrent infection following the application of modular silver-coated knee arthrodesis prosthesis [21,27,36,37,38,39,40,41,42,43] and to compare it with those of other modular implants. The studies and the referred outcomes of knee arthrodesis after the application of both silver-coated and not modular implants on infected total knee arthroplasties are summarized in Table 2. 

Our results showed that MRSA was the primary cause for septic failure of TKA being in line with the international literature reporting that *Staphylococcus* spp. is the leading microorganism related with the infection of knee reconstructions, resulting in increased failure rates [21,27,36,37,38,39,40,41]. It is worth noting that even if the risk for infection recurrence of TKA is not clearly defined, it tends to be lower (even 26%) including with Stanmore or custom-made arthrodesis modules [21,42,44]. Contrariwise, slightly greater risk (14.3%) for re-infection after the arthrodesis implantation has been detected by Rao, et al. [36] and Iacono, et al. [37]. Although the comparison between these case series and our research is difficult due to population heterogeneity and implant differences, our findings were in line with the outcomes being reported by the above studies. Moreover, Βarlett et al. who studied the mid-term results after the application of Stanmore knee arthrodesis prosthesis in 10 patients (including a patient suffering from angiosarcoma) with a mean follow-up of 56.4 months (range, 15–199 months), reported eradication of infection in 90% of the patients. Angelini et al. examined the application of modular endoprosthesis (MUTARS-Munster Arthrodesis implant Implant cast, Germany) for knee arthrodesis in 32 patients (seven with a failed TKA accompanied by segmental bone loss, inadequate soft tissue coverage and extensor mechanism deficiency, and 25 with a major bone defect after tumour resection) reporting similar effectiveness rates in the eradication of the infective agents. In the above study, silver-coated prostheses were applied in only two-patients. 

According to our results, infection recurrence has been reported in one patient (14%), who was finally treated with above the knee amputation and currently remains free of symptoms. Moreover, in the final implantation surgery of two patients (28%), positive intraoperative cultures (*Staphylococci* spp.) were observed, leading to the administration of intravenous antibiotics for 2 weeks. However, at the last follow up they remain free of infection or other implant associated complications. We must highlight the fact that, to the best of our knowledge, this is the first study that examined the clinical and functional outcomes of knee fusion after the application of silver coated modular mega-endoprostheses in the treatment of PJI, so far. Silver is a promising material for implant coating, and it seems to prevent bacterial colonization and periprosthetic infections [45,46,47,48,49,50]. Specifically silver ions can bind to bacterial wall proteins and to DNA, destroying the bacterial cellular wall or preventing microbe protein synthesis and bacterial colonisation [24,25]. Moreover, silver ions can result in intracellular production of reactive oxygen molecules that are toxic for the bacteria. Indeed, preclinical trials of animal experimental models demonstrated that the application of silver coated implants in rabbits was associated with significantly lower rates of infection compared with those with titanium implants (7% versus 47%) [51]. However, the exact role of silver coated implants used for knee arthrodesis following PJIs has not been fully elucidated yet. Wilding et al. examined the application of silver-coated arthrodesis nails in eights patients with known PJI and reported two cases of superficial and periprosthetic reinfection, respectively [27]. Moreover, the use of a custom-made device, by a Waldemar Link, based on the Mega C prostheses system (with the addition at the titanium non-articulating surfaces of an innovative silver coating composed of two layers) in 33 patients with septic complication or at high risk for infection was evaluated by Scoccianti et al. [52]. The results of the above study showed that the recurrence rate among the patients with known previous infection was only 9.5% [52]. Additionally, successful knee arthrodesis with eradication of the infection has been reported in an immunosuppressed patient with chronic *Aspergillus osteomyelitis* without detecting any silver-coated associated adverse events [53]. Consequently, the use of silver coating is a promising alternative method to reduce the infection rates of tumour prostheses or arthrodesis devices.

The survivorship of the modular arthrodesis implant may vary and depends on the type of complications and the infectious agent. In our study, the survivorship rates of the modular silver-coated arthrodesis system were estimated at 86% during a follow up time (minimum) of 24 months. According to Angelini et al., the survivorship of the modular endoprostheses was 50% at five years and 25% at ten years [44]. As the above study included both oncologic and non-oncologic patients, further multivariate analysis on prostheses did not demonstrate significant difference between the patients with tumour resection, the presence of infection or a gastrocnemius flap after a follow-up period of 9.5 years. Finally, even if the infection was complicated in 1/3 of the patients, the survivorship of the implant seems to be unaffected. 

Knee arthrodesis can be used as a salvage procedure following complicated revision arthroplasty and presents superior functional outcomes when compared to amputation [1]. In most of the cases, these patients could ambulate independently with the assistance of a walking aid and displayed superior functional levels on pain indices compared to the patients after amputation. The patients included in the present study represented a difficult-to-treat population because they underwent prosthetic knee arthrodesis for unrevivable TKA after previous extensive unsuccessful interventions to eradicate periprosthetic infection. The MUTARS silver-coated prosthesis was selected for these patients to maintain mobility and to avoid amputation. Six out of eight patients were treated successfully with good functional results (median OKS value 34.6 ± 6.4), experienced mild or no pain symptoms (median VAS value 2.4 ± 0.9) and remained free of infection during their last follow up. Although our results agree with the functional outcomes reported by Rao [36], Iacono [37] and Scarponi et al. [39], it must be noted that many studies have described unchanged knee function, or patient dissatisfaction following this surgical procedure [21,27,38,43]. Postoperative calculation of leg length discrepancy displayed that the affected leg was 2.07 ± 0.68 cm shorter than the unaffected, but all the patients were able to perform everyday activities with the use of cane. Leg length discrepancy was directly related to patients’ satisfaction, and it was similar between the studies that examined modular knee arthrodesis implants (Mean: 10 ± 10 mm, Min: 5, Max: 34 mm) as it was reported by Putman et al. [38]. After meticulous bone and soft tissue debridement, the application of silver-coating implant is linked to construct stability and early weight bearing making this technique a promising alternative for limb salvage.

Finally, in our cases the clinical symptoms of argyria or peripheral neuropathy were not observed. Our results are in consistence with the findings of Scoccianti et al. [52] who did not report symptoms of silver-associated side effects. Moreover, the above study identified low levels of serum silver ranging from 2.09 to 5.33 μg/L after two weeks and 36 months, respectively. Similarly, low circulating silver levels were also referred to by the study of Hardes et al. [49], ranging from 1.93 to 12.98 μg/L at the third and 24th post-operative month, respectively. These results may explain not only the long-lasting antimicrobial activity of silver coated prosthesis but the limited local and systemic clinical adverse effects of their application. 

We acknowledge that, despite the encouraging mid-term results, the study has some limitations. It is a retrospective study, with a small number of patients, without a comparison group. However, all eight patients had persistent periprosthetic joint infection, extensive bone loss and extensor tendon deficiency and were operated by the same surgical team and their follow-up and functional results were evaluated by the same investigator at the time of the latest follow-up, providing the necessary accurate measurements, adequate analysis and correct interpretation for the increased validity of our results [54,55]. Randomized international multicentred studies with a larger number of patients are needed to establish the promising results of this knee arthrodesis technique.

## 5. Conclusions

- Knee arthrodesis is an important alternative to amputation in infected and multiple revised TKA.
- It provides functional improvement and eradication of the infection.
- The application of silver-coated knee arthrodesis implants is associated with increased implant survivorship as well as with satisfactory mid-term clinical and patient-reported outcomes as the limb salvage procedure for failed infected TKA with significant bone loss and extensor tendon deficit.

## Figures and Tables

**Table 1:** Patients’ demographic characteristics and postoperative clinical outcomes.

Patient ID	Gender	Age (Years)	BMI (kg/m^2^)	Soft Tissue Coverage	Follow-Up (Months)	Clinical Outcome	Limb Length Discrepancy (cm)	VAS (0–5)	OKS
1	M	66	24	Adequate	59	Success–Supression	2	3	42
2	M	73	27	Adequate	24	Success	1.5	1	24
3	F	62	29	Gastrocnemius flap	36	Failure	3	3	47
4	F	70	27	Adequate	27	Success–Supression	3	3	44
5	M	57	31	Gastrocnemius flap	24	Success	2	2	31
6	F	72	30	Adequate	29	Success	1.5	2	28
7	F	75	22	Adequate	25	Success	1.5	1	27
8	F	61	27.5	Adequate	26	Success	1.5	2	27

BMI: Body Mass Index, VAS: Visual Analogue Scale, OKS: Oxford Knee score.

**Table 2:** Main studies on knee arthrodesis with modular implants following PJIs.

Study	Country	Number of pts *	Age (y.o)	Infecting Organism Isolated	Type of Modular Arthrodesis Prosthesis	Outcomes
Rao et al. 2009 [36]	United Kingdom	7	72.3 (64–86)	2 pts with *S. aureus*, Staphylococcus sciuri, Coag negative staph, 2 pts with more than one organisms	Endo-Model^®^ Knee Fusion Nail (Newsplint, UK/Waldemar Link^®^, GmbH & Co. KG, Hamburg, Germany)	4 pts with complete relief from their pain, VAS pain score improved from a mean of 7.9 (7.5–9.2) pre-operatively to 1.5 (0–6.4) postoperatively, 2 pts required revision (the first due to fracture in cement mantle of the femoral component, the second due to infection and insufficient fracture of the femoral shaft), infection recurrence 14.3%
Bartlett et al. 2011 [21]	United Kingdom	10 ***	51.5 (35–70)	Not Applicable	Stanmore knee arthrodesis (Stanmore Implants Worldwide, Middlesex, UK)	In total, 1 pt died 24 months post-op (not related to procedure), 1 pt above the knee amputation, 1 pt with fracture of the growing mechanism shaft. Among 9 pts with PJI, 1 pt with recurrent infection, 1 pt with wound dehiscence, independently immobilize, improvement of pain
Iacono, et al. 2012 [37]	Italy	22	69.3 (53–85)	Not Applicable	Link nail (Link Laboratory, Hamburg, Germany	3 pts with recurrent infection -(2 of whom underwent amputation), 8 of the rest 8 patients (38.09%) with no pain (VAS = 0), 5 pts (23.8%) with score of 1, 3 pts (14.28%) with score of 2, 1 (4.7%) scored 3, 1 pt with 8 score (mean VAS 1.2), 1 intraoperative shaft fracture, 90% of the pts satisfied with the result. Infection recurrence 14.3%
Putman, et al. 2013 [38]	France	31	67 (48–80)	*S. aureus* in 10 cases (including 1 case with methicillin-resistant SA [MRSA]), coagulase-negative *Staphylococcus* (CNS) in six cases (including 1 with methicillin-resistant CNS [MRCNS]). In 7 (22.5%) pts several organisms	Customized dual-component arthrodesis nail (Link EndoModelTM, Boves, France)	In 3 pts sinus tracts with recurrent clinical infection, required revision surgery, residual pain without recurrent infection in 14 patients, dissatisfaction in 22 pts, 3 of them died more than 24 months after the knee arthrodesis. None of the pts required amputation for uncontrolled infection. 67% bone union achieved. 26 % recurrent infection(required revision)
Scarponi, et al. 2014 [39]	Italy	38	65 (46–82)	*S. aureus* in 19 cases, 10 with MRSA), coagulase-negative *Staphylococci* in 12 patients (five methicillin resistant), *Streptococcus* spp. in 5 pts, *P. aeruginosa* in 3, *Enterobacter* spp. In 3, *Escherichia coli* in 3, *Enterococcus faecalis* in 3	Endo-Model^®^ Knee Fusion Nail SK Modular System^®^, Waldemar-Link, Hamburg, Germany	At a two-year follow-up, 4 pts with infection recurrence, 2 of whom underwent above-knee amputation. 2 of the rest with pain in the operated leg at rest, 20 pts without or very mild pain, 9 with moderate pain and 5 with more severe pain.
Galluser, et al. 2015 [40]	Switzerland	15	67 (42–87)	Not Applicable	Wichita Fusion Nail^R^	Mean follow up of 33 months. 9 with primary fusion (75%), 3 pts needed a surgical revision for non-union or wound dehiscence, 1 with peroneal nerve palsy. Infection rate 0%
Wilding, et al. 2016 [27]	United Kingdom	8	64.9 (55–75)	7/8 organism isolation prior arthrodesis, 3 with polymicrobial, 1 pt with rifampicin resistant strain of gram positive *Staphylococcus*	RS Arthrodesis 90 Implant, Implantcast, Buxtehude, Germany	1 pt lost to follow up, 37.5% post-op complications, 2 pt with re-operation recurrent infection (1 with superficial collection, improvement in pain, mobilization from seated, no change in kneeling (inability pre-operatively)
Faure, et al. 2020 [41] **	France	31	67 (48–80)	62% same bacteria during revisions (5 of 8 cases with revision)	Customized dual-component arthrodesis nail (Link EndoModelTM, Boves, France)	Median follow-up of 158 months (138–163), no mechanical failures (implant breakage or aseptic loosening). 8 pts (26%) undergo revision surgery (all had infections) (2 since 2012). In 5 pts (16%) implants change, 3 pts (10%) need debridement and lavage before suppressive antibiotics. 16 % incidence of implant change and surgical revision at 10 years. 16 pts died in total.
Mayes, et al. 2020 [42]	USA	15	68.5 (45–85)	None in 4 cases, one pathogen in 8 cases (*P.mirabilis*, Oxacillin-resistant *Staphylococcus aureus*, Group B *Streptococcus*, *P. aeruginosa*, *S. epidermidis*, *Enterobacter cloacae Enterobacter cloacae*, *Streptococcus*), more than 1 in 2 cases	14 pts * with Stryker (Kalamazoo, MI) GMRS system, 1 pt with a Biomet (Warsaw, IN) Orthopedic Salvage System modular KF	2 of the pts died postoperatively (one immediately from pulmonary embolism, due to cement embolization, one opted for AKA (unhappy with the result), none of the pts * with clinical signs of persistent or recurrent infection, and no one on suppressive antibiotics.
Stavrakis, et al. 2022 [43]	USA	81	67 (45–84)	*S. epidermidis* most common organism (22.2% of cases), 18.5% with *S. aureus* (60% methicillin-sensitive *Staphylococcus aureus*, 40% methicillin-resistant *Staphylococcus aureus*). 11% of patients with multiorganism infection, 11.1% culture-negative	OSS Modular Arthrodesis System, Zimmer Biomet, Warsaw, IN	17% with persistent infection or reinfection, 7.4 % of them underwent above knee amputation, knee function unchanged based, did get worse clinically. Over 80% of pts underwent reimplantation and endoprosthetic reconstruction

* patient, ** same cohort as Putman, et al. [38], evaluated after 11.5 years of follow-up, *** study on 9 patients with revision for PJI/1 with primary surgery for angiosarcoma.

## Data Availability

The data presented in this study are available on request from the corresponding author. The data are not publicly available due to privacy restrictions.
